# Investigating the Influence of Body Mass Index on Organs at Risk Doses for Adjuvant High-Dose-Rate Vaginal Cuff Brachytherapy in Patients with Early-Stage Endometrial Carcinoma: A Single-Center Experience

**DOI:** 10.3390/diagnostics15070795

**Published:** 2025-03-21

**Authors:** Alexandra Timea Kirsch-Mangu, Diana Cristina Pop, Alexandru Țipcu, Andrei-Rareș Avasi, Claudia Ordeanu, Ovidiu Florin Coza, Alexandru Irimie

**Affiliations:** 1 Department of Oncology, “Iuliu Hatieganu” University of Medicine and Pharmacy, 400347 Cluj-Napoca, Romania; alexandru.tipcu@elearn.umfcluj.ro; 2 “Prof. Dr. I. Chiricută” Oncology Institute, 400015 Cluj-Napoca, Romania; patcasdiana@yahoo.com (D.C.P.); avasiandrei@yahoo.com (A.-R.A.); claudia_ordeanu@yahoo.com (C.O.); coza.ovidiu@iocn.ro (O.F.C.)

**Keywords:** endometrial cancer, body mass index, vaginal cuff brachytherapy, three-dimensional vaginal cuff brachytherapy, dosimetry, obesity

## Abstract

**Background**: Endometrial cancer is the most common gynecologic malignancy in developed countries, with obesity recognized as a major risk factor contributing to its incidence. The rising prevalence of obesity has significant implications for treatment planning, particularly in radiation therapy approaches such as high-dose-rate (HDR) vaginal cuff brachytherapy, which is commonly used as adjuvant therapy in early-stage endometrial carcinoma. Body Mass Index (BMI) is a key factor in brachytherapy, as increased adiposity may alter dosimetric parameters, affecting radiation distribution and doses received by organs at risk (OARs). Understanding the correlation between BMI and radiation dose to OARs is essential for optimizing treatment planning and minimizing adverse effects. Identifying dose variations across different BMI categories may help refine patient-specific brachytherapy approaches to ensure both efficacy and safety. **Objectives**: This study aims to investigate the influence of Body Mass Index (BMI) on the doses received by organs at risk (OAR) during high-dose-rate (HDR) vaginal cuff brachytherapy in patients diagnosed with early-stage endometrial carcinoma. Understanding the relationship between BMI and OAR doses could enhance treatment planning and minimize complications. **Methods**: We collected brachytherapy data for 242 endometrial cancer patients treated with adjuvant HDR vaginal cuff brachytherapy. The patients were categorized based on their BMI into normal weight, overweight, and obese groups. Dosimetric data were collected for OARs, including the bladder, rectum, and sigmoid colon, and also for dose fractionation, D90%, and the active length of the brachytherapy cylinder. The analysis included comparing the doses received by each organ across different BMI categories using appropriate statistical methods. **Results**: Preliminary findings indicated a significant variation in the doses to OARs correlating with BMI classifications. Obese patients exhibited slightly higher mean doses to the rectum and sigmoid compared to those with a normal BMI. The statistical analysis demonstrated that as BMI increased, the dose to these organs at risk also tended to increase, suggesting a need for adjusted treatment planning strategies in this population. **Conclusions**: Obesity is a key concern in endometrial cancer patients, with higher BMI linked to slightly increased doses to the rectum and sigmoid, though treatment remained homogeneously delivered. Future prospective clinical studies are essential to explore the relationship between these dosimetric findings, specifically the correlation between higher BMI, increased doses to organs at risk (OARs), and late treatment-related toxicities. This research is needed to better understand the long-term implications and to optimize therapeutic outcomes.

## 1. Introduction

Endometrial cancer (EC) is the most common gynecological malignancy in developed countries and the sixth most common cancer in women worldwide. Reflecting the trends seen in other Eastern European countries, the incidence of endometrial cancer has been on the rise in Romania [1].

In 1983, Bokhman developed the initial subtype categorization of endometrial carcinoma, differentiating between Type I and Type II endometrial malignancies based on clinical and hormonal characteristics. Type I endometrial carcinoma, generally known as the endometrioid type, constitutes 80–90% of all sporadic endometrial malignancies. Type II EC, or non-endometrioid tumors, comprise the remaining 10–20% of sporadic endometrial tumors [2].

The traditional dualistic model of endometrial cancer classifies tumors into Type I (endometrioid, estrogen-dependent, typically low-grade) and Type II (non-endometrioid, estrogen-independent, aggressive subtypes such as serous and clear cell carcinoma). However, the 2023 FIGO classification (Table 1) [3] refines the staging system, emphasizing prognostic factors and integrating molecular characteristics to guide management.

The incorporation of The Cancer Genome Atlas (TCGA) [3] molecular subtypes—POLE-ultramutated, microsatellite instability-high (MSI-H), copy-number low (no specific molecular profile), and copy-number high (p53-abnormal)—has revolutionized risk stratification and personalized treatment approaches. This molecular framework influences therapeutic decisions, with POLE-mutated tumors demonstrating excellent prognosis and often requiring de-escalated therapy, while p53-abnormal carcinomas benefit from more aggressive multimodal treatment. The updated treatment algorithm now integrates molecular profiling alongside traditional clinicopathologic parameters to optimize adjuvant therapy, reflecting a shift towards precision oncology in endometrial carcinoma management.

Approximately 70–90% of Type I (estrogen-dependent) EC patients are obese [4]. Factors such as urbanization, lifestyle changes, and increasing obesity rates contribute to the rise in endometrial cancer cases, as patients often present with obesity, diabetes, and hypertension.

Endometrial cancer was the first type of cancer identified as associated with obesity, a condition that not only promotes cancer development but also negatively impacts treatment outcomes. Women with severe obesity who develop endometrial cancer face a significantly higher risk of mortality from both the cancer and related co-morbidities compared to their lean counterparts. Premenopausal women account for up to 14% of cases, mostly as a result of elevated body mass index (BMI). Patients with a BMI of >40 were more likely to have endometrioid histology, lower-stage disease, and lower-grade tumors than women with a BMI of <30 [4].

Obesity has also been linked to lower quality of life among endometrial cancer survivors, as well as a higher body mass index (BMI). This association has been well established and follows a dose–response relationship, with the incidence of endometrial cancer increasing as body mass index (BMI) increases [5].

A meta-analysis of 26 research papers conducted by the American Institute for Cancer Research indicated that with each increment of five BMI units, the probability of developing endometrial cancer escalated by 50% [6].

Calculating BMI at diagnosis helps categorize patients into risk groups based on their obesity status (overweight, obese, or morbidly obese). Higher BMI often correlates with a poorer prognosis and can influence clinical decisions regarding treatment intensity.

The main treatment for endometrial cancer patients is surgery (total abdominal hysterectomy and bilateral salpingo–oophorectomy) with or without pelvic lymph node dissection or sentinel lymph node sampling followed by adjuvant treatment [7].

The recommendations for adjuvant therapy in early-stage endometrial cancer are based on FIGO staging (The International Federation of Gynecology and Obstetrics) with regard to the presence or absence of various risk factors, including older age, extensive myometrial invasion, high tumor grade, large tumor size, and lymphovascular space invasion (LVSI) [8,9].

The integration of molecular subtypes into conventional endometrial cancer management has revolutionized treatment strategies, moving towards precision oncology. The four molecular subtypes identified by The Cancer Genome Atlas (TCGA)—POLE-ultramutated, microsatellite instability-high (MSI-H), no specific molecular profile (NSMP), and p53-abnormal—now guide risk stratification and therapeutic decisions [3].

The classification of early-stage uterine cancer into low-risk, intermediate-risk, and high-risk categories relies on a combination of these risk factors; however, definitions can vary among researchers and studies.

In early-stage endometrial cancer, the vaginal cuff is the most common site for recurrence when adjuvant treatment is not used. Most recurrences take place in this location or in the proximal third of the vagina, with only about 10% occurring in the middle and distal thirds [10]. Depending on the surgical technique, the scar region may cover 1 cm or even more, and the potentially affected vaginal wall can have a thickness of 2–5 mm, depending on the patient’s age. Since the vaginal cuff is the most common site of relapse for this type of cancer, based on clinical evidence, adjuvant brachytherapy is widely considered the standard treatment to decrease vaginal recurrence in patients with intermediate-risk EC after surgery.

The Postoperative Radiation Therapy in Endometrial Carcinoma (PORTEC-2) trial [10] was initiated to determine whether vaginal brachytherapy (VCBT) would be as effective as external beam radiation therapy (EBRT) in reducing vaginal recurrence while also causing fewer treatment-related side effects and enhancing quality of life. Patients were randomized post-surgery to receive adjuvant radiation by either pelvic EBRT or VCBT, showing no significant difference in vaginal cuff recurrences or overall survival between the two treatment regimens. The results of this randomized trial established that VCBT is highly effective at achieving local control and minimizing the risk of vaginal recurrence, which is the most common site of disease recurrence in patients with high–intermediate-risk endometrial carcinoma. VCBT provides excellent vaginal control and rates of locoregional recurrence, with overall survival and disease-free survival rates comparable to EBRT. Additionally, VCBT exhibits a significantly higher quality of life and reduced gastrointestinal toxicity when compared to EBRT. Therefore, VCBT should be the preferred adjuvant treatment for patients with high-intermediate-risk endometrial carcinoma [11]. The result of this study has translated into an increasing utilization of high-dose-rate (HDR) VCBT as the treatment of choice in high–intermediate-risk patients based on various society-specific guidelines for the management of patients with EC.

There are various fractionation schemes used clinically, with no general consensus on the superiority of one regimen over another. The American Brachytherapy Society (ABS) does not necessarily recommend one fractionation scheme over another in their report [12].

One of the most important surveys of vaginal brachytherapy practices [13] revealed that the most frequently utilized fractionation scheme is 7 Gy × 3 fractions, prescribed to a depth of 0.5 cm, followed by 6 Gy × 5 fractions prescribed to the vaginal surface. The next most common schemes were 5.5 Gy × 4 fractions and 5 Gy × 5 fractions, both prescribed to a depth of 0.5 cm, with 7.5 Gy × 5 fractions prescribed to the vaginal surface last, with the total dose prescribed being in the 30 Gy margin. It is important to note that there is significant variability in the dose-fractionation. All these regimens appear effective based on institutional reports. Acceptable and frequently utilized fractionation schemes are presented in Table 2.

Another important problem regarding VCBT is the prescription depth of the dose. The common prescription depth of 5 mm from the cylinder surface, though potentially more toxic than prescribing to the surface, can be justified by the location of vaginal lymphatics [17,18]. Ninety-five percent of the vaginal lymphatics lie within 3 mm from the vaginal surface, so ensuring an adequate dose to at least this depth may be important [19].

Brachytherapy is preferably started 4–6 weeks after surgery. Care must be taken that the surgical scar at the vaginal vault has healed sufficiently, which usually takes at least 3 weeks.

Applicator selection is determined through a clinical vaginal examination using a speculum and/or valves. In most patients, the postoperative vagina assumes a cylindrical shape and can be effectively managed with a vaginal cylinder. Furthermore, vaginal cylinders are advantageous for a constricted vagina.

The choice depends on several factors, including the anatomy of the vaginal cuff, the patient’s post-surgical status, and the desired dose coverage. The diameter of the cylinder should be as large as comfortably possible to minimize mucosal dose and improve dose homogeneity. A proper fit ensures good surface contact, preventing air gaps that could lead to dose inhomogeneity. It is imperative for the vaginal mucosa to be in contact with the applicator surface to allow for an effective dose distribution.

It is also important to establish the length of the vagina on the same gynecological examination prior to the treatment in order to prescribe the dose to the correct length. The length of the applicator should be chosen to adequately cover the target volume while avoiding unnecessary irradiation of the healthy vaginal mucosa.

Although post-hysterectomy vaginal brachytherapy is a simple treatment technique, imaging with the applicator in place should be performed to verify and document the size and position of the applicator and to determine the dose to the organs at risk (OAR) and optimize dose planning [20].

Proper applicator selection is essential to achieving effective local control while reducing the risk of complications such as vaginal stenosis or mucosal ulceration.

A correct gynecological examination ensures a standardized initial approach, after which each patient receives a personalized treatment plan tailored to her specific anatomy. The selection of the vaginal cylinder applicator for endovaginal brachytherapy in endometrial cancer is crucial to ensure optimal dose distribution while minimizing toxicity to the surrounding organs. An individual applicator is insufficient for addressing all anatomical variations and disease manifestations. Various applicators are necessary to address these diverse presentations.

The required applicators at a given institution are contingent upon the patient case mix, the clinical expertise, and the preferences of the radiation oncologist.

The ABS recommends that institutions should have vaginal cylinders available in various lengths and diameters (ranging from 1.5 to 4 cm) to treat a variety of patients [21].

In PORTEC-2, the upper half of the vagina was treated, while Sorbe et al. focused on treating the upper two-thirds of the vagina. The ABS guidelines recommend administering vaginal cuff brachytherapy (VCBT) with an active length (AL) of 3–5 cm after hysterectomy [22]. Practices vary widely, but the most frequently used fixed length and fractional length prescriptions for endometrial cancer are 4 cm and the proximal half of the vagina, respectively. For clear cell and serous histologic features, treatment of the entire vagina should be considered [12].

In addition to the different fractionation schedules, other risk factors can also influence the toxicities and the treatment outcomes, and as such, there is a shortage of research examining the impact of obesity on dosimetric factors regarding VCBT. The aim of our study was to assess whether BMI influences the dosimetric factors in VCBT treatment planning. To accomplish this, we identified two primary objectives:

(1) To assess and evaluate the correlation between higher BMI and the width and active length of the applicator. This information would improve the preparation process for patient treatment, including education and applicator selection. If a higher BMI correlates with a wider applicator and a longer active length, the treatment duration could be nearly twice as long compared to patients with a normal BMI.
(2) To analyze whether a higher BMI affects the doses delivered to the target volume and organs at risk (OARs). Ultimately, we aim to determine whether an elevated BMI is associated with increased doses to OARs, which may subsequently lead to a higher rate of late toxicities. Furthermore, this could imply that a higher BMI may require a more personalized approach to managing late toxicities.

We conducted a literature review across major databases (PUBMED, Medline, and Scopus), and our findings revealed only two articles aligned with our study objectives, both of them published over 10 years ago. The first one, by Boyle et al. [23], included results from only 30 patients, and no information regarding applicator width; moreover, they included in their analysis patients who underwent EBRT as well. The second article, from Sabater et al., included only 59 patients with both endometrial and cervical cancer and they also did not include any analysis regarding the applicators used. To this end, we sought to investigate variables that were not included in their study design, especially for a significantly larger cohort of patients treated exclusively with adjuvant vaginal cuff brachytherapy. Because of our larger cohort of patients compared with the previously published studies, we think that our results would add value to treatment advantages or disadvantages.

## 2. Materials and Methods

Between February 2016 and May 2024, our department treated 377 patients who required exclusive vaginal cuff brachytherapy as an adjuvant treatment following surgery. However, from this group, we selected only those patients who received 3D (three-dimensional) image-guided brachytherapy, excluding 135 patients who had undergone 2D (two-dimensional) treatment. In 2D brachytherapy, volumetric analysis is not possible, as it relies solely on planar imaging (X-rays) without 3D anatomical visualization. Since our study focuses on volumetric assessment, including these cases would not align with our study objectives, making them unsuitable for our cohort.

Three-dimensional brachytherapy, as recommended by international guidelines, employs CT or MRI-based imaging, allowing for the direct visualization of the tumor or surgical scar and surrounding structures, leading to more accurate dose calculation and individual treatment adaptation. Three-dimensional image-guided brachytherapy enables precise contouring of the tumor and OARs, resulting in optimized dose distribution. Given these differences, including both 2D and 3D brachytherapy patients in our study would introduce significant heterogeneity in terms of treatment planning, dosimetry, and clinical outcomes. This would compromise the validity and reliability of our results.

As our study specifically evaluates the benefits of 3D brachytherapy, we chose to exclude 2D patients to maintain a homogeneous study cohort and align with the current European guidelines that discourage the use of 2D brachytherapy in modern clinical practice.

So, the final analysis was performed only on 242 patients treated exclusively with 3D- CT image-guided adjuvant vaginal cuff brachytherapy.

We collected body metrics (weight and height in order to calculate BMI) and brachytherapy data in a retrospective manner from patients included in the study.

Patients’ characteristics regarding age, staging, prescribed dose, cylinder diameter and prescribed length, D2cc (minimum dose within the 2 cm^3^ volume receiving the highest dose) to organs at risk (OARs), and dose to target volume D90% HR_CTV (dose to 90% of high-risk clinical target volume) are shown in (Table 3). In terms of histological EC subtype, all the included patients were diagnosed with endometrioid adenocarcinoma.

To ensure optimal reproducibility and the lowest dose to organs at risk, all patients underwent an at-home bowel preparation and had a Foley balloon inserted at the time of brachytherapy.

A gynecological examination was made prior to the treatment in order to assess

- The vaginal length and width of the vaginal vault, ensuring a good fit of the cylinder.
- Surgical alterations: post-hysterectomy changes, vaginal stenosis, or fibrosis can influence the choice.
- The choice of size of the cylinder (diameter: 2–4 cm), which depends on patient anatomy (vaginal width and length).
- Patient tolerance: comfort and feasibility of the procedure play a role in determining which applicator can be safely used.

Applicator insertion was performed using the largest diameter cylinder that could comfortably fit into the vaginal vault to guarantee tight mucosal apposition and cover the target volume, with the cylinder positioned to remain parallel to the cranio-caudal axis of the patient, and this was evaluated with imaging after insertion. We used three types of applicators that were available in our department from Varian Medical Systems (Palo Alto, Santa Clara, CA, USA), respectively, the closed CT vaginal cylinder (available since 2016 in our department), the shielded cylinder (available since 2018 in our department), and the universal stump cylinder (available since 2022 in our department). Applicators were chosen regarding patient characteristics, availability, and different diameter sizes.

For contouring of the target volume, physicians included a 5 mm volume from the cylinder surface to the tip, while the length of the contour was based on the patient’s characteristics of vagina length, usually 3 to 5 cm. Organs at risk, respectively, bladder, rectum, sigmoid, and small bowel, were contoured according to GEC-ESTRO guidelines [24].

With each insertion, supplementary factors that may have affected individual dosimetry, including cylinder diameter, active length, and dose regimens, were recorded.

Body metrics data were collected at the time of hysterectomy, and BMI was computed using the conventional formula: mass (kg) divided by the square of the height (m^2^). Patients were then categorized into four groups based on their BMI according to the World Health Organization (WHO) classification [25].

### 2.1. CT Acquisition

All patients underwent a planning CT scan on a General Electric Discovery RT scanner at first insertion, utilizing 2.5 mm thick slices while in head-first supine position. At each fraction, a Foley bladder catheter was employed to instill a dilute contrast medium (2 mL of Omnipaque 350 mixed with 48 mL of saline solution), while the Foley balloon was filled with saline solution to enhance bladder visibility during segmentation and to ensure volume reproducibility during treatment.

Holloway et al. conducted a retrospective study indicating that the inter-fraction variance for D2cc doses to the bladder and rectum is minimal, at 6–8% [26]. They concluded that there is insufficient evidence to justify the need for recording doses to organs at risk (OARs) with each fraction. While the delivery of VCBT in multiple sessions based on a single plan raised concerns, data indicated that a certain level of homogeneity between insertions was necessary [27]. This led to the development of well-established pre-insertion protocols for rectum and bowel preparation, as well as bladder filling [28].

### 2.2. Segmentation and Planning

CTs were transferred to Varian Medical Systems (Palo Alto, Santa Clara, CA, USA) 3D Brachytherapy Planning (we used version 13.6 until 2019, version 16.0 until 2023, and version 17.0 ever since) for contouring and treatment planning. For treatment planning, we utilized a nominal source strength of 10 Ci, 40,700 cGy cm^2^/h for a 192 Iridium source and employed the TG-43 line-source dose calculation formalism. Treatment delivery was carried out based on the actual source strength at the moment of administration, added automatically at the machine console. Also, for target volume and organs at risk dose evaluation, we used the well-known EQD2 spreadsheet from Nag S [29].

The entire bladder volume was outlined, and the rectum was defined as extending from 2 cm above the cylinder tip to 2 cm below the last target volume contoured slide.

After ensuring a minimal dose of 100% to target volume, doses were reported according to ICRU [30] and GEC-ESTRO recommendations [24], respectively, for the dose to 90% of target volume (D90% of HR-CTV) and for organs at risk, the dose to maximally exposed 2 cm^3^ of OARs (D2cc). All the doses were extracted from the treatment planning and DVH and converted to biological equivalent dose using the EQD2 table by Nag S. Cylinder size, active length and dose prescription were also recorded.

### 2.3. Body Mass Index Assessment

As stated before, calculating BMI at diagnosis helps categorize patients into risk groups based on their obesity status; therefore, the assessment of BMI was made at the time of hysterectomy for all patients included in the study. Regarding the 6-week gap between surgery and brachytherapy, to our knowledge, there are publications that state any modifications in BMI value between treatments.

BMI was calculated and classified according to the World Health Organization (WHO) definitions [25]. The formula for BMI is weight in kilograms divided by the square of the height in meters. The categories are as follows: underweight < 18.5 kg/m^2^; normal weight 18.5–24.9 kg/m^2^; overweight 25.0–29.9 kg/m^2^; obese class I 30.0–34.9 kg/m^2^; obese class II 35.0–39.9 kg/m^2^; and obese class III > 40.0 kg/m^2^.

### 2.4. Statistical Analysis

Data collection was conducted via the Microsoft Office 365 Suite—Office Excel. Data processing, statistical analysis, and chart preparation were conducted utilizing International Business Machines Corporation^®^ Statistical Product and Service Solutions^®^ v.26.0. (IBM Corporation, Armonk, NY, USA).

Distribution analysis for the continuous data was performed using Kolmogorov–Smirnov and Shapiro–Wilk tests. Different groups were compared using either Mann–Whitney U or Kruskal–Wallis H (with Dunn-Bonferroni post hoc) tests, depending on the number of groups involved. Linear relationships were assessed using Spearman’s correlations. We included the potential predictors for OAR doses in a series of multiple linear regression models. Model fit was assessed by the ANOVA *p*-value. All tests were considered statistically significant for a Pearson’s *p* = 0.05. All performed testing was two-tailed. The output of this statistical analysis includes *p*-values, a graph of groups, group comparisons, R^2^, and residual plots.

## 3. Results

Given the fact that most of our data would be continuous in nature, we conducted a power analysis suited to a linear regression in order to determine an adequate lot size. For an alpha of 0.05 and beta of 0.2, 199 patients should be included in order to detect a 0.2 size, medium type-f effect.

In the selected period, we identified 242 endometrial cancer patients treated surgically with salpingo-oophorectomy with or without lymph node staging, and, based on the surgical findings from the histopathological exam, they underwent adjuvant exclusive vaginal cuff brachytherapy as subsequent treatment, as per our institution protocol. BMI at the time of hysterectomy was recorded.

Regarding the applicator choice, it was related to the patient’s anatomy and applicator availability (we did not have all of them available in 2016 when we started using 3D CT brachytherapy). The important part of applicator choice was for it to fit perfectly into the patient’s vaginal vault without any air between the applicator and the mucosa of the vagina, even though we used different types and different widths of cylinders. From a dosimetric perspective, we successfully achieved our planning goals of ensuring that 100% of the dose covered the target volume while sparing adjacent organs at risk.

For all patients, mean height was 165 cm [150–188], mean weight was 82 kg [47–135], and mean BMI was 30.27 [19.5–52.7]. We found significant weak positive relationships between BMI and age (*p* = 0.032).

A total of 242 planning CT scans were assessed at first insertions, which translated into 242 treatment plans for a total of 945 insertions. Dose schedules were 7 Gray (Gy) × 3 fractions (fr), 5 Gy × 5 fr, 6 Gy × 4 fr, and 5 Gy × 4 fr, with 54.5% of all treatments being the 7 Gy × 3 fr schedule and 44.21% the 5 Gy × 5 fr, leaving 1.2% for the other schedules. Dose regimens reflected a biological equivalent dose of ~30 Gy prescribed to target volume. All treatment plans had a minimum dose % of 100, and according to planning calculations, the D90% (EQD2) dose to target volume ranged from 28.7 to 45.7 Gy with a mean dose of 36.38 Gy.

We analyzed the distribution of different BMI classes regarding the different brachytherapy dose regimens. We found significant frequency variation (*p* = 0.042). We also observed a slight shift in proportion for the 7 Gy × 3 dose regimen (increasingly less used for more obese categories) and the 5 Gy × 5 dose regimes (increasingly more used for more obese categories).

**Figure 1:**
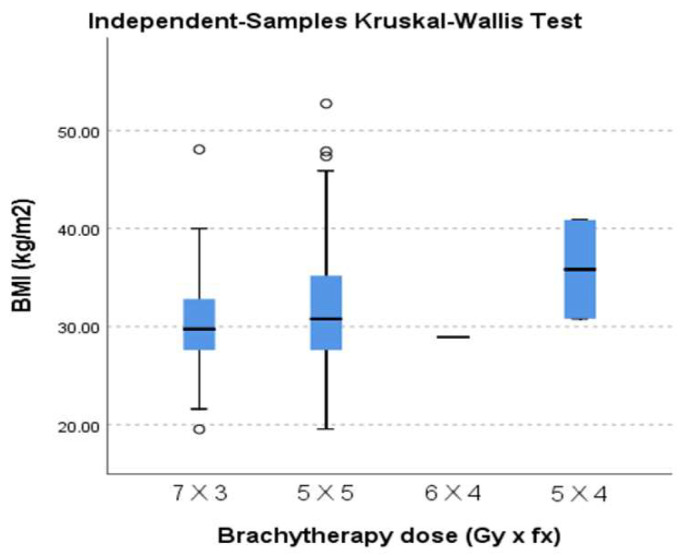
Dose variability for different BMI.

We tested whether there was any variability between the different brachytherapy dose regimens prescribed regarding median BMI, and we found no statistically significant difference (*p* = 0.302). In this matter, the treatment was delivered homogeneously, even though we used different dose schedules (Figure 1).

**Figure 2:**
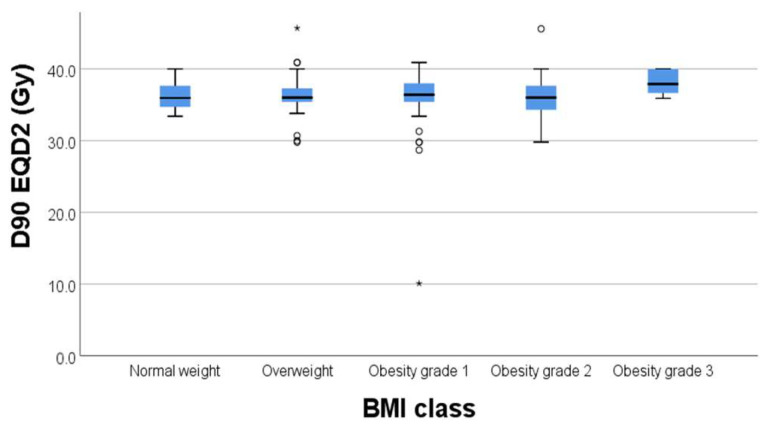
Box plot of D90% and BMI class. The line represents the median, the box the interquartile range, and the whiskers represent range. The outliers in each group were represented outside the boxplot whiskers by either circles (outliers out of the 1.5 times the interquartile range) or stars (*) (outliers out of the 3.0 times the interquartile range).

Regarding the correlation between BMI and D90% to target volume, we did not find any statistically significant correlations with *p* = 0.231 (Figure 2.) In this regard, BMI did not significantly affect the dose to the target volume (D90%).

**Figure 3:**
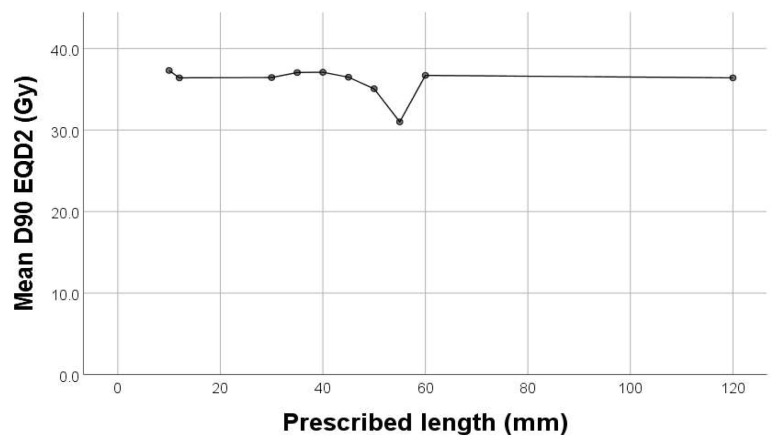
Correlation between D90% and cylinder diameter.

In our study, there was a significant weak negative correlation between D90% (Gy) and cylinder diameter (*p* = 0.013, rho = −0.159) and prescribed length (*p* < 0.001, rho = −0.255) (Figure 3). The outliers in each group were represended outside the boxplot whiskers by either circles (outliers out of the 1.5 times the interquartile range) or stars (outliers out of the 3.0 times the interquartile range).

For organs at risk, we extracted from the DVH the 2cc dose and used the EQD2 Nag S. spreadsheet for dose calculations. As results, we had mean D2cc for bladder 19.62 Gy [4.2–31.7], rectum 20.56 Gy [8.6–26.3], sigmoid 6.32 Gy [1.2–8.6], and small bowel 6.32 Gy [0.4–7.8].

**Figure 4:**
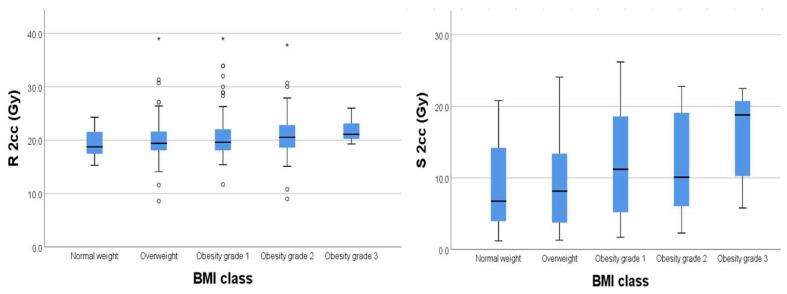
Box plot of rectum and sigmoid for each BMI class. The outliers in each group were represented outside the boxplot whiskers by either circles (outliers out of the 1.5 times the interquartile range) or stars (*) (outliers out of the 3.0 times the interquartile range).

We found significant weak positive relationships (Figure 4) between BMI and D2cc for rectum (*p* = 0.003) and D2cc for sigmoid (*p* = 0.004), respectively.

**Figure 5:**
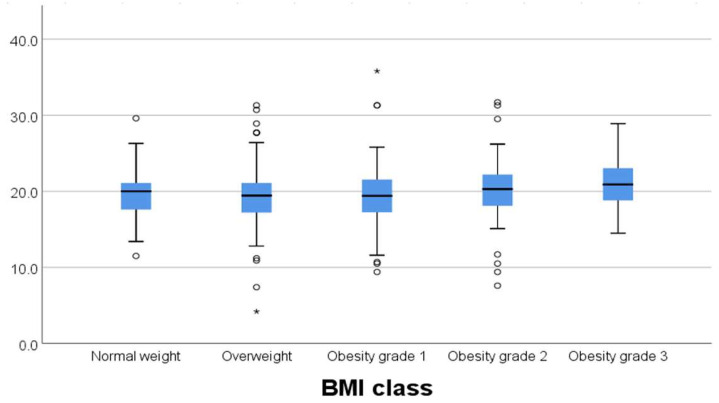
Box plot of bladder dose for each BMI class. The outliers in each group were represented outside the boxplot whiskers by either circles (outliers out of the 1.5 times the interquartile range) or stars (*) (outliers out of the 3.0 times the interquartile range).

Regarding the bladder, there was no significant correlation between BMI and D2cc (*p* = 0.182) (Figure 5).

**Figure 6:**
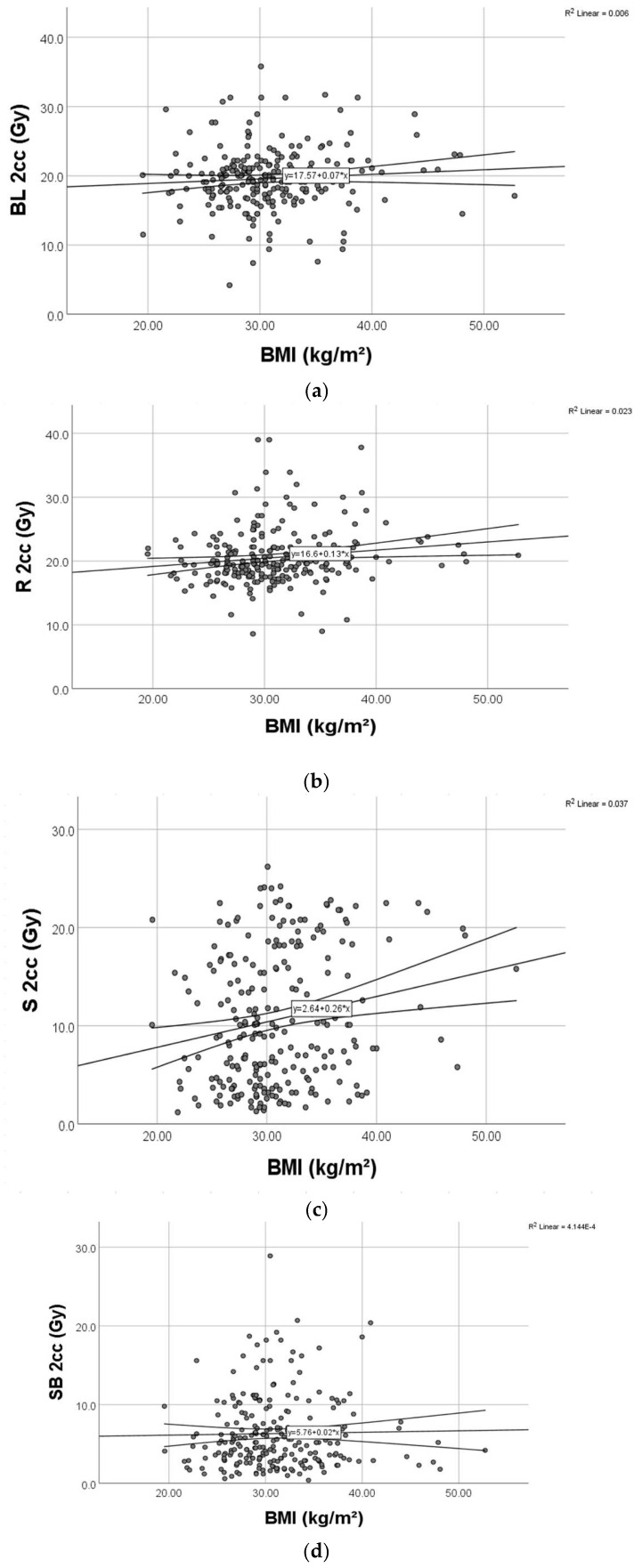
(**a**) Scatterplot for bladder dose and BMI. (**b**) Scatterplot for rectum dose and BMI. (**c**) Scatterplot for sigmoid dose and BMI. (**d**) Scatterplot for small bowel dose and BMI.

We generated a series of multiple linear regression models, one for each OAR dose in question, including every possible predictor variable (BMI, age, tumor grade, cylinder diameter, and prescribed length). The ANOVA *p* and adjusted R^2^ for each of the four OARs regression models were as follows: bladder (*p* = 0.084, adj R^2^ = 0.02), rectum (*p* = 0.217, adj R^2^ = 0.009), sigmoid (*p* = 0.04, adj R^2^ = 0.028), and small bowel (*p* = 0.999, adj R^2^ = 0.02). As also seen in Table 4a–d, BMI remains an independent predictor for rectum and sigmoid doses. The B-value, along with the 95% confidence intervals, can be observed in the respective tables. For each 1 unit increase in BMI, we observed an increase of 0.085 Gy in bladder dose, 0.129 Gy increase in rectum dose, 0.244 Gy increase in sigmoid dose, and 0.02 Gy increase in small bowel dose (Figure 6).

## 4. Discussions

Vaginal cuff brachytherapy treatment planning has traditionally followed guidelines established by the International Commission on Radiation Units and Measurements, with doses to organs at risk (OARs) historically limited to 2D points. However, over the past decade, the widespread availability of 3D imaging has enabled CT-based treatment planning, providing volumetric dose information for both OARs and the target volume.

Given that 98.8% of the patients in our study were over 50 years old and considering obesity as a national health concern, we aimed to assess whether BMI influences treatment outcomes. As a first step, we evaluated its impact on treatment doses. Despite BMI not being a recognized risk factor in brachytherapy decision-making, its relevance in the Romanian healthcare landscape prompted our investigation. Additionally, all patients underwent surgery at our hospital, allowing easy access to BMI data from medical records. BMI remained stable during the 6–8-week interval between surgery and brachytherapy.

Our findings reveal a statistically significant but weak positive correlation between BMI and age (*p* = 0.032), indicating a slight tendency for BMI to increase with age. Boyle et al. [23] reported that women with lower BMI tend to have reduced abdominal adipose tissue, potentially exposing normal tissue to higher radiation doses. In our dataset, high BMI did not significantly correlate with dose to the target volume, suggesting consistent treatment homogeneity among patients. However, a higher BMI was associated with slightly increased doses to OARs, particularly the rectum and sigmoid, where we found a statistically significant correlation (*p* = 0.003 and *p* = 0.004, respectively). This suggests that potential dose increases in obese patients may necessitate mitigation strategies. Boyle et al. also highlighted that the high mobility of the sigmoid colon could contribute to these variations. Furthermore, increased adipose tissue may alter radiation penetration, reducing treatment efficacy and potentially leading to OAR overdose due to tissue inhomogeneity.

**Figure 7:**
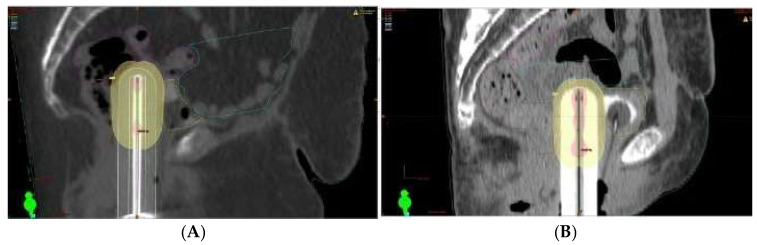
(**A**) Sagittal image for patient with 38.2 BMI, 95.8% dose to bladder, 88.6% dose to rectum, and 91.4% dose to sigmoid. (**B**) Sagittal image for patient with 23.8 BMI, 95.6% dose to bladder, 46.6% dose to rectum, and 13.4% dose to sigmoid.

To better highlight the dose differences in the rectum and sigmoid, we selected a patient with a high BMI (38.2) and one with a low BMI (23.8). The images show the contouring of the organs at risk, with brown for rectum and magenta for sigmoid. For these two patients, the percentage of the dose recorded for the bladder was approximately the same (approximately 95%), while for the rectum and the sigmoid, the differences in the dose recorded were 42% and 78%, respectively (Figure 7).

Unlike Sabater et al. [28], who focused only on the bladder and rectum, our study examined all OARs currently considered in endometrial brachytherapy in relation to BMI. Regarding dose schedules, two patients initially prescribed 5 Gy × 5 underwent only four fractions (5 Gy × 4) due to refusal of the last treatment session. The majority received one of the most common regimens for exclusive VCBT—either 7 Gy × 3 or 5 Gy × 5—where we observed a significant correlation between dose schedule and BMI.

The distribution of staging varied notably across different BMI categories, with a slight tendency towards higher FIGO stages in patients with elevated BMI. Furthermore, our data revealed a weak negative correlation between the dose to the target volume and both applicator width (*p* = 0.013) and active length (*p* < 0.001), indicating that appropriate applicator selection contributed to sufficient target coverage.

In terms of dose fractionation and OAR exposure, we found that sigmoid and small bowel doses at 2 cc were lower in patients treated with the 7 Gy × 3 schedule. Since BMI showed a statistically significant correlation with 2cc doses to the rectum and sigmoid, further confirmed by multiple linear regression analysis, we conclude that BMI is an independent predictor of OAR doses in vaginal cuff brachytherapy.

## 5. Conclusions

While BMI has been extensively studied in relation to endometrial cancer incidence and prognosis, its impact on brachytherapy dosimetry remains less explored. Our study contributes by quantifying BMI-related variations in organ-at-risk (OAR) doses and emphasizing the need for clinical adaptation.

Recognizing BMI as a factor influencing dose distribution supports the need for patient-specific treatment planning, including applicator selection, insertion technique, and adaptive dose optimization, particularly in patients with higher BMI. Although our study did not assess direct clinical toxicity outcomes, prior research links even modest OAR dose increases to higher rates of late toxicity, such as rectal bleeding, fistula formation, and vaginal stenosis. Our findings suggest that targeted interventions—such as improved applicator positioning, individualized dose constraints, or pre-treatment weight management—could help mitigate these risks.

The observed dose variations highlight the need for future studies integrating dosimetric data with clinical toxicity outcomes to refine brachytherapy protocols for patients with high BMI. By identifying modifiable factors influencing dosimetry, our study lays the groundwork for potential protocol adaptations, though direct recommendations require further validation.

While obesity is a well-established concern in endometrial cancer, our study reinforces its impact on brachytherapy by demonstrating that higher BMI correlates with increased radiation doses to the rectum, sigmoid, and small bowel. However, the D90%—a key dosimetric parameter—was not significantly affected, suggesting adequate target volume coverage. The increased OAR doses in obese patients may lead to a higher risk of toxicities, such as radiation cystitis and proctitis, which can significantly affect quality of life and necessitate personalized management strategies.

Despite being a single-institution study, the large sample size and detailed statistical analysis provide valuable insights that could influence future clinical practices. Further research is needed to explore the relationship between higher BMI, increased OAR doses, and late treatment-related toxicities. Large-scale, multi-institutional trials and meta-analyses will be essential for validating these findings and optimizing treatment protocols. Given the limited existing data, our study strengthens the understanding of obesity as a risk factor in adjuvant vaginal cuff brachytherapy (VCBT) and underscores the need for prospective research to refine treatment strategies accordingly.

This study does not propose immediate protocol modifications, but it provides clinically relevant data on BMI-related dosimetric changes in brachytherapy. These findings highlight the importance of individualized patient assessment and encourage further research into how treatment planning can be refined for patients with higher BMI.

## Figures and Tables

**Table 1:** FIGO 2023 staging for endometrial carcinoma.

Stage	Substage	Description
Stage I	IA	Tumor limited to the endometrium or invading less than half of the myometrium.
Stage I	IB	Tumor invades equal to or more than half of the myometrium.
Stage II		Tumor invades cervical stroma but does not extend beyond the uterus. Includes endocervical glandular involvement with stromal invasion.
Stage III	IIIA	Tumor involves the serosa and/or adnexa.
Stage III	IIIB	Tumor involves the vagina and/or parametria.
Stage III	IIIC	Tumor involves regional lymph nodes.
Stage III	IIIC1	Pelvic lymph node metastasis.
Stage III	IIIC2	Para-aortic lymph node metastasis.
Stage IV	IVA	Tumor invades the mucosa of the bladder or rectum.
Stage IV	IVB	Distant metastases, including intra-abdominal metastases and/or involvement of inguinal lymph nodes.

**Table 2:** Fractionation schedules for vaginal cuff brachytherapy. ^a^ PORTEC-2 [10], ^b^ ABS survey, also equivalent to ^f^ [12,13], ^c^ Michigan series [14], ^d^ Sorbe et al. [15], ^e^ MDAC series, ^f^ Australian series [16], and ^g^ DFCI series [17].

Prescription Point	Dose per Fraction	Number of Fractions
0.5 cm depth from vaginal surface	7 ^a^	3
	5.5 ^b^	4
	5 ^c^	5
	2.5 ^d^	6
Vaginal surface	6 ^e^	5
	8.5 ^f^	4
	4 ^g^	6

**Table 3:** Patient characteristics (numerical data expressed as mean with median in brackets; Spearman’s correlations were performed to assess proportionality). N/A: Not Applicable.

Variables	All Patients	Normal Weight	Overweight	Obese gr1	Obese gr2	Obese gr3	*p*-Value	Spearman’s rho
Age (y)	66.22 (66.22)	66.29 (67)	64.72 (65.04)	66.79 (67.86)	68.1 (67.68)	69.59 (69.86)	0.032	0.138
Stage								N/A
FIGO IA	52 (21.5%)	0 (0%)	29 (29%)	18 (22.8%)	4 (11.1%)	1 (9.1%)	<0.001	N/A
FIGO IB	185 (76.4%)	16 (100%)	71 (71%)	58 (73.4%)	32 (88.9%)	8 (72.7%)	<0.001	N/A
FIGO II	5 (2.1%)	0 (0%)	30 (0%)	3 (3.8%)	0 (0%)	2 (18.2%)	<0.001	N/A
Dose schedule								N/A
7 Gy × 3 fr	132 (54.5%)	7 (43/8%)	62 (62%)	45 (57%)	16 (44.4%)	2 (18.2%)	0.042	N/A
5 Gy × 5 fr	107 (44.2%)	9 (56.3%)	37 (37%)	33 (41.8%)	20 (55.6%)	8 (72.7%)	0.042	N/A
6 Gy × 4 fr	1 (0.4%)	0 (0%)	1 (1%)	0 (0%)	0 (0%)	0 (0%)	0.042	N/A
5 Gy × 4 fr	2 (0.8%)	0 (0%)	0 (0%)	1 (1.3%)	0 (0%)	1 (9.1%)	0.042	N/A
Cylinder diameter (mm)	32.26 (30)	31 (30)	32.11 (30)	32.33 (35)	33.33 (35)	31.45 (30)	0.166	0.089
Prescribed length (mm)	42.59 (40)	42.63 (45)	42.6 (40)	41.84 (40)	43.75 (42.5)	44.09 (40)	0.546	0.039
BL 2cc (Gy)	19.62 (19.6)	19.66 (20)	19.32 (19.45)	19.51 (19.4)	20.2 (20.3)	21.11 (20.9)	0.182	0.086
R 2cc (Gy)	20.56 (19.6)	19.26 (18.75)	21.12 (19.4)	20.91 (19.6)	21.2 (20.55)	21.84 (21.1)	0.003	0.193
S 2cc (Gy)	10.66 (9.3)	8.78 (6.75)	9.06 (8.15)	11.8 (11.2)	11.89 (10.1)	15.84 (18.8)	0.004	0.187
SB 2cc (Gy)	6.32 (5.15)	5.05 (3.85)	6.14 (5.2)	6.98 (5.6)	5.77 (5.1)	6.93 (4.2)	0.754	0.02
D90 EQD2 (Gy)	36.38 (36.3)	36.23 (35.95)	36.32 (36.36)	36.36 (36.4)	36.1 (36)	38.22 (37.9)	0.231	0.077

**Table 4:** (a) Multiple regression model for bladder D2cc (Gy). (b) Multiple regression model for rectum D2cc (Gy). (c) Multiple regression model for sigmoid D2cc (Gy). (d) Multiple regression model for small bowel D2cc (Gy).

(a)	Unstandardized Coefficients		Standardized Coefficients	t	Sig.	95.0% Confidence Interval for B	
	B	Std. Error	Beta			Lower Bound	Upper Bound
(Constant)	20.347	4.251		4.787	0.000	11.973	28.722
BMI (kg/m^2^)	0.085	0.056	0.098	1.517	0.131	−0.025	0.194
Age (y)	−0.090	0.034	−0.169	−2.612	0.010	−0.157	−0.022
Tumor grade	0.344	0.393	0.057	0.875	0.382	−0.430	1.117
Cylinder diameter (mm)	0.026	0.088	0.019	0.293	0.770	−0.148	0.199
Prescribed length (mm)	0.027	0.034	0.050	0.779	0.437	−0.041	0.095
Dependent variable: BL 2cc							
**(b)**	**Unstandardized Coefficients**		**Standardized Coefficients**	**t**	**Sig.**	**95.0% Confidence Interval for B**	
	**B**	**Std. Error**	**Beta**			**Lower Bound**	**Upper Bound**
(Constant)	−6.719	6.554		−1.025	0.306	−19.631	6.194
BMI (kg/m^2^)	0.244	0.086	0.182	2.835	0.005	0.074	0.413
Age (y)	0.050	0.053	0.061	0.951	0.343	−0.054	0.155
Tumor grade	0.423	0.605	0.045	0.699	0.485	−0.770	1.616
Cylinder diameter (mm)	0.151	0.136	0.071	1.110	0.268	−0.117	0.418
Prescribed length (mm)	0.020	0.053	0.025	0.385	0.701	−0.084	0.125
Dependent variable: S 2cc							
**(c)**	**Unstandardized Coefficients**		**Standardized Coefficients**	**t**	**Sig.**	**95.0% Confidence Interval for B**	
	**B**	**Std. Error**	**Beta**			**Lower Bound**	**Upper Bound**
(Constant)	6.610	4.531		1.459	0.146	−2.317	15.537
BMI (kg/m^2^)	0.020	0.059	0.022	0.340	0.734	−0.097	0.137
Age (y)	−0.007	0.037	−0.013	−0.192	0.848	−0.079	0.065
Tumor grade	0.005	0.419	0.001	0.012	0.990	−0.819	0.830
Cylinder diameter (mm)	−0.005	0.094	−0.003	−0.052	0.959	−0.190	0.180
Prescribed length (mm)	−0.007	0.037	−0.012	−0.190	0.850	−0.079	0.065
Dependent variable: SB 2cc							
**(d)**	**Unstandardized Coefficients**		**Standardized Coefficients**	**t**	**Sig.**	**95.0% Confidence Interval for B**	
	**B**	**Std. Error**	**Beta**			**Lower Bound**	**Upper Bound**
(Constant)	17.587	4.185		4.203	0.000	9.342	25.831
BMI (kg/m^2^)	0.129	0.055	0.153	2.355	0.019	0.021	0.237
Age (y)	−0.003	0.034	−0.007	−0.101	0.920	−0.070	0.063
Tumor grade	0.120	0.387	0.020	0.310	0.757	−0.642	0.881
Cylinder diameter (mm)	0.022	0.087	0.017	0.258	0.797	−0.149	0.193
Prescribed length (mm)	−0.041	0.034	−0.078	−1.206	0.229	−0.108	0.026
Dependent variable: R 2cc							

## Data Availability

The data presented in this study are available on request from the corresponding author. The data are not publicly available due to patient privacy.
